# A novel method for isolation of histones from serum and its implications in therapeutics and prognosis of solid tumours

**DOI:** 10.1186/s13148-017-0330-x

**Published:** 2017-03-29

**Authors:** Divya Reddy, Bharat Khade, Riddhi Pandya, Sanjay Gupta

**Affiliations:** 1 Epigenetics and Chromatin Biology Group, Gupta Lab, Cancer Research Institute, Advanced Centre for Treatment, Research and Education in Cancer (ACTREC), Tata Memorial Centre, Kharghar, Navi, Mumbai, 410210 MH India; 2 Homi Bhabha National Institute, Training School Complex, Anushakti Nagar, Mumbai, MH 400085 India

**Keywords:** cNUC, Histones, Serum, Cancer, HDACs, Diagnosis

## Abstract

**Background:**

Dysregulation in post-translational modifications of histones and their modifiers are now well-recognized as a hallmark of cancer and can be used as biomarkers and potential therapeutic targets for disease progression and prognosis. In most solid tumours, a biopsy is challenging, costly, painful or potentially risky for the patient. Therefore, non-invasive methods like ‘liquid biopsy’ for analysis of histone modifications and their modifiers if possible will be helpful in the better clinical management of cancer patients.

**Methods:**

Here, we have developed a cost-effective and time-efficient protocol for isolation of circulating histones from serum of solid tumor, HCC, called Dual Acid Extraction (DAE) protocol and have confirmed by mass spectrometry. Also, we measured the activity of HDACs and HATs in serum samples.

**Results:**

The serum purified histones were profiled for changes in histone PTMs and have shown a comparable pattern of modifications like acetylation (H4K16Ac), methylation (H4K20Me3, H3K27Me3, H3K9Me3) and phosphorylation (γ-H2AX and H3S10P) to paired cancer tissues. Profiling for the histone PTM changes in various other organs of normal and tumor bearing animal suggests that the changes in the histone PTMs observed in the tumor serum is indeed due to changes in the tumor tissue only. Further, we demonstrate that the observed hypo-acetylation of histone H4 in tissue and serum samples of tumor bearing animals corroborated with the elevated HDAC activity in both samples compared to normal. Interestingly, human normal and tumor serum samples also showed elevated HDAC activity with no significant changes in HAT activity.

**Conclusions:**

Our study provides the first evidence in the context of histone PTMs and modifiers that liquid biopsy is a valuable predictive tool for monitoring disease progression. Importantly, with the advent of drugs that target specific enzymes involved in the epigenetic regulation of gene expression, liquid biopsy-based ‘real time’ monitoring will be useful for subgrouping of the patients for epi-drug treatment, predicting response to therapy, early relapse and prognosis.

## Background

Histones are well conserved basic proteins which associate to form an octameric core around which the DNA is wrapped to form a nucleosome [1]. The N-terminal tails of histones protrude out of the nucleosome and undergo a variety of post-translational modifications (PTMs) like acetylation, phosphorylation, methylation, sumoylation and ubiquitination. The occurrence and functioning of these PTMs are an orchestrated event of ‘writers’ (adds modifications), ‘readers’ (recognizes modification) and ‘erasers’ (remove the modifications). Histone PTMs dynamically maintain the chromatin states, and thus any deregulation may lead to altered gene expression as observed in diseases like cancer [2]. Indeed, the loss of histone H4 lysine 16 acetylation (H4K16Ac) and lysine 20 trimethylation (H4K20Me3) are considered as hallmark of most human cancers [3]. Similarly, global H3 and H4 hypo-acetylation have been proven to correlate with tumour phenotype, prognostic factors and patient outcome in breast and prostate cancers [4, 5]. Decades of research have discovered a battery of histone PTMs that are altered in cancer and are now referred as ‘histone onco-modifications’, but none has reached clinics primarily due to technological limitations in the diagnosis of solid tumours.

Traditionally, cancer diagnosis and staging of solid tumours are done with an imaging technique followed by a surgical biopsy. But biopsy, being invasive, requires a complex setting and well-trained clinician and is occasionally difficult and risky for some advanced stage patients. Therefore, diagnosis or monitoring of solid tumours utilizing circulating epigenetic biomarkers in blood samples, if possible, will prove to be a very powerful tool and will overcome all the earlier limitations. Indeed, research conducted over the last few years has identified and detected epigenetic biomarkers associated with cancer, including aberrant DNA methylation patterns, miRNA profiles and histone signatures in body fluids of the patients [6, 7].

The levels of circulating nucleosomes (cNUCs) and histones are found to be elevated in a number of disease conditions like inflammation caused by bacterial infections, autoimmune diseases like SLE, stress and trauma [8]. cNUCs are released into the blood by apoptotic cells during these processes [9]. Elevated levels of cNUCs have been reported in lung, breast, colorectal, renal and gastric cancer compared to patients with inflammation and healthy individuals [10]. In case of gastrointestinal tumours, a positive correlation between cNUC levels, tumour stage and metastasis has been established [11]. In case of advanced non-small cell lung carcinoma and cervical cancer, a correlation between clinical outcome in response to chemotherapy and cNUCs has been observed [12]. On circulating DNA, hypo-methylation pattern has been established in CRC patients [13]. Histone modifications like H3K9me3 and H4K20me3 have been detected on cNUCs [14]. In another study, the ratio of H3K9me3/nucleosome and H4K20me3/nucleosome was found to be less in serum of breast and colorectal cancer patients compared to healthy individuals [15]. Though studies on histone PTMs on cNUCs have revealed interesting aspects, but how similar is circulating epigenetic signature to solid tumour signatures in terms of their histone PTMs and their modifiers has not been established yet. This especially is important as because any changes in the tissue epigenetic signature, if possible, can be read via the use of serum histones that can act as a good prognosis and a diagnostic marker.

Here, we used a cost-effective method named as dual acid extraction (DAE) method, to isolate pure histones from serum with the aim to understand the correlation between circulating histone PTM with that of solid tumour (hepatocellular carcinoma, HCC) tissues in animal model. Furthermore, we for the first time measured the histone acetylase (HAT) and histone deacetylase (HDAC) activities in serum samples of normal and solid tumour-bearing animals and human patient samples.

## Methods

### Animal handling and experiments

All the experiments were performed on male Sprague-Dawley rats (spp. *Rattus norvegicus*) after approval of the Institute Animal Ethics Committee (IAEC# 04/2014), Advanced Centre for Treatment Research and Education in Cancer and the Committee for the Purpose of Control and Supervision on Animals, India standards. The detailed protocol to induce liver carcinogenesis is as previously described [16]. Tissue samples (liver, lung, kidney and brain) were fixed in formalin and prepared as paraffin-embedded blocks according to standard protocols. The H&E-stained sections were microscopically reviewed for histopathological alterations to confirm normal and HCC. Post-anaesthesia, the blood was slowly collected by cardiac puncture (preferably from ventricle) from 120 days NDEA (late stage liver cancer)-treated animals after which they were sacrificed [17]. For early stage liver cancer, blood from the tail vein was collected from 90 days NDEA-treated animals.

### Human blood sample collection and serum isolation

Blood samples of 24 cancer patients were collected retrospectively from the Tumor Tissue Repository (TTR) of Advanced Centre of Treatment, Research and Education in Cancer (ACTREC), Tata Memorial Centre after Ethical Approval from Institute Ethics Committee III (Project number 164) along with six healthy adult human volunteers. As the samples were collected retrospectively, institute ethics committee III approved waiver of consent for working on patient samples. Whole blood from patients was collected prior to surgery, allowed to clot at room temperature for 15–30 min. The clot was then removed by centrifugation at 5000 rpm for 10 min at 4 °C. The resulting supernatant (serum) is transferred into multiple tubes to avoid freeze and thaw cycles and finally stored in liquid nitrogen containers in ACTREC, TMH-TTR. The samples collected were from 2013 to 2016 with confirmed histopathological tumour type as mentioned in Table 2.

### Isolation of histones from serum

Total proteins were precipitated from the serum (5 ml) by the slow addition of Trichloroacetic acid (TCA) to a final concentration of 20% with continuous and vigorous mixing. The precipitation was allowed to carry out on the ice for 30 min, followed by centrifugation at 15,000 rpm for 15 min. The obtained protein pellet was homogenized with glass teflon homogenizer in three volumes (*w*/*v*) of 0.2 M sulphuric acid (H_2_SO_4_), till the pellet was completely dispersed and a milky white liquid is formed, which then was intermittently vortexed for >2 h at 4 °C. This mixture was then centrifuged at 16,000 rpm for 20 min at 4 °C. To the supernatant obtained post-centrifugation, four volumes of acetone was added and histones were precipitated overnight at −20 °C. The pellet obtained post-centrifugation at 16,000 rpm for 20 min at 4 °C was washed twice with two volumes of each of acidified acetone and acetone twice. The histone pellet was air dried and eventually suspended in 50 μl of 0.1% β-mercaptoethanol in H_2_O and stored at −20 °C. Histone concentrations were determined by Bradford method of protein estimation. Protein standards were prepared containing a range of 0 to 5 μg of bovine serum albumin in 5 ml of 1× Bradford reagent. Histone samples were also prepared similarly. Samples were vortexed and incubated at room temperature for 5 min. Absorbance was measured at 595 nm and the blank was adjusted. Histone samples were estimated for protein concentration by plotting a standard curve.

### Isolation of histones from liver tissue

Histones were extracted and purified as described earlier [18]. Briefly, liver tissues (1 g) were homogenized in 10 ml lysis buffer (15 mM Tris-Cl pH 7.5, 60 mM KCl, 15 mM NaCl, 2 mM EDTA, 0.5 mM EGTA, 0.34 M sucrose, 0.15 mM β-mercaptoethanol, 0.15 mM spermine and 0.5 mM spermidine) with 1× protease inhibitor cocktail and phosphatase inhibitor cocktail. Nuclei were then isolated by sucrose gradient centrifugation. The three volumes of homogenate was layered on top of one volume of 1.8 M sucrose. Nuclei pellet obtained by centrifugation at 26,000 rpm for 90 min at 4 °C was resuspended in 0.2 M H_2_SO_4_ and incubated for >2 h at 4 °C with intermittent vortexing. After centrifugation at 16,000 rpm for 20 min at 4 °C, the histones were precipitated at −20 °C for overnight with addition of four volumes of acetone to the supernatant. Post-centrifugations at 16,000 rpm for 20 mins at 4 °C, pelleted histones were air dried and suspended in 0.1% β-mercaptoethanol in H_2_O and stored at −20 °C.

### Resolution and analysis of histones

The purified histones from serum and tissue that were resolved on 18% sodium duodecyl sulphate-polyacrylamide gel electrophoresis (SDS-PAGE) was either stained by silver staining method [19] or transferred to PVDF membrane, probed with site-specific modified histone antibodies, against H4K16Ac (Millipore#07-329), H4K20Me3 (Abcam#9053), γH2AX (Millipore#05-636), H3S10P (Millipore#06-570), H3K27Me3 (Millipore#07-449) and H3K9Me3 (Abcam#8898), and signals were detected by ECL plus detection kit (Millipore #WBKLS0500). Gel loading equivalence was done by silver staining method.

### Analysis of histones by LC-MS

The purified histones (15 μg) were subjected to in-solution trypsin (20 ng/μl) digestion in 50 mM ammonium bicarbonate, pH 8.0 at 37 °C for 2 h (1:200 enzyme:substrate) as described previously [20]. Enzymatic digestion was stopped by adding 10% trifluoroacetic acid (TFA) to a final pH <3. Peptides were then desalted with ZipTip C18 columns (Millipore) and lyophilized prior to analysis by LTQ Orbi-trap-MS/MS (ABSCIEX). The MS instrument was operated in the data‐dependent mode to automatically switch between full scan MS and MS/MS acquisition. Mascot generic format (mgfs) files were generated and were searched against protein database using Mascot version 2.3 (Matrix Science, London, UK).

### HDAC and HAT activity assays

Assays were performed using the colorimetric HDAC and HAT activity assay kits from BioVision (BioVision Research Products, USA) according to manufacturer’s instructions. Briefly for HDAC activity assay, 50 μl of serum from normal, early and late tumours were diluted in 85 μl of H_2_O; then, 10 μl of 10× HDAC assay buffer were added followed by addition of 5 μl of the colorimetric substrate; samples were then incubated at 37 °C for 1 h. Subsequently, the reaction was stopped by adding 10 μl of lysine developer and left for additional 30 min at 37 °C. Samples were then read in an ELISA plate reader at 405 nm. Each sample was also treated with 2 μl (12 μM final concentration) of Trichostatin A (TSA) for 10 min before performing HDAC activity assay, these readings are also plotted and labelled with TSA, alongside the readings for serum samples not treated with TSA (W/O TSA). Experiments were performed in triplicates and average absorbance was plotted.

For HAT activity assay, 50 μl of serum was diluted in 80 μl H_2_O (final volume), and for background reading, 80 μl H_2_O was added in the reaction instead of the sample. Then, 50 μl of 2× HAT assay buffer was added followed by addition of 5 μl of each of the colorimetric substrates 1 and 2 and 8 μl NADH-generating enzyme. The samples were incubated at 37 °C for 1 to 4 h depending on the colour development. Subsequently, samples were read in an ELISA plate reader at 440 nm. Experiments were done in triplicates and average absorbance was plotted.

### Statistical analysis

All numerical data were expressed as average of values obtained ±standard deviation (SD). Statistical significance was determined by conducting paired Student’s *t* test.

## Results

### Isolation of serum histones

**Fig. 1:**
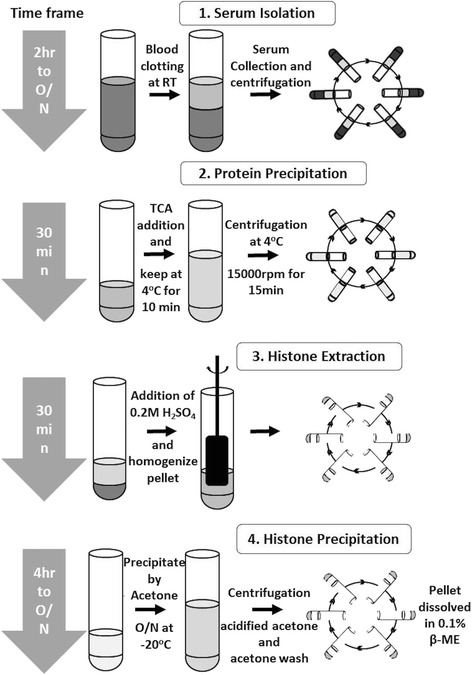
Diagrammatic representation of protocol for isolation of histones from blood. The dual acid extraction (DAE) protocol involves four crucial steps: (1) serum isolation from blood; (2) total protein precipitation from serum by trichloroacetic acid; (3) histone extraction from precipitate by sulphuric acid and (4) precipitation, washing and dissolution of extracted histone precipitation

**Fig. 2:**
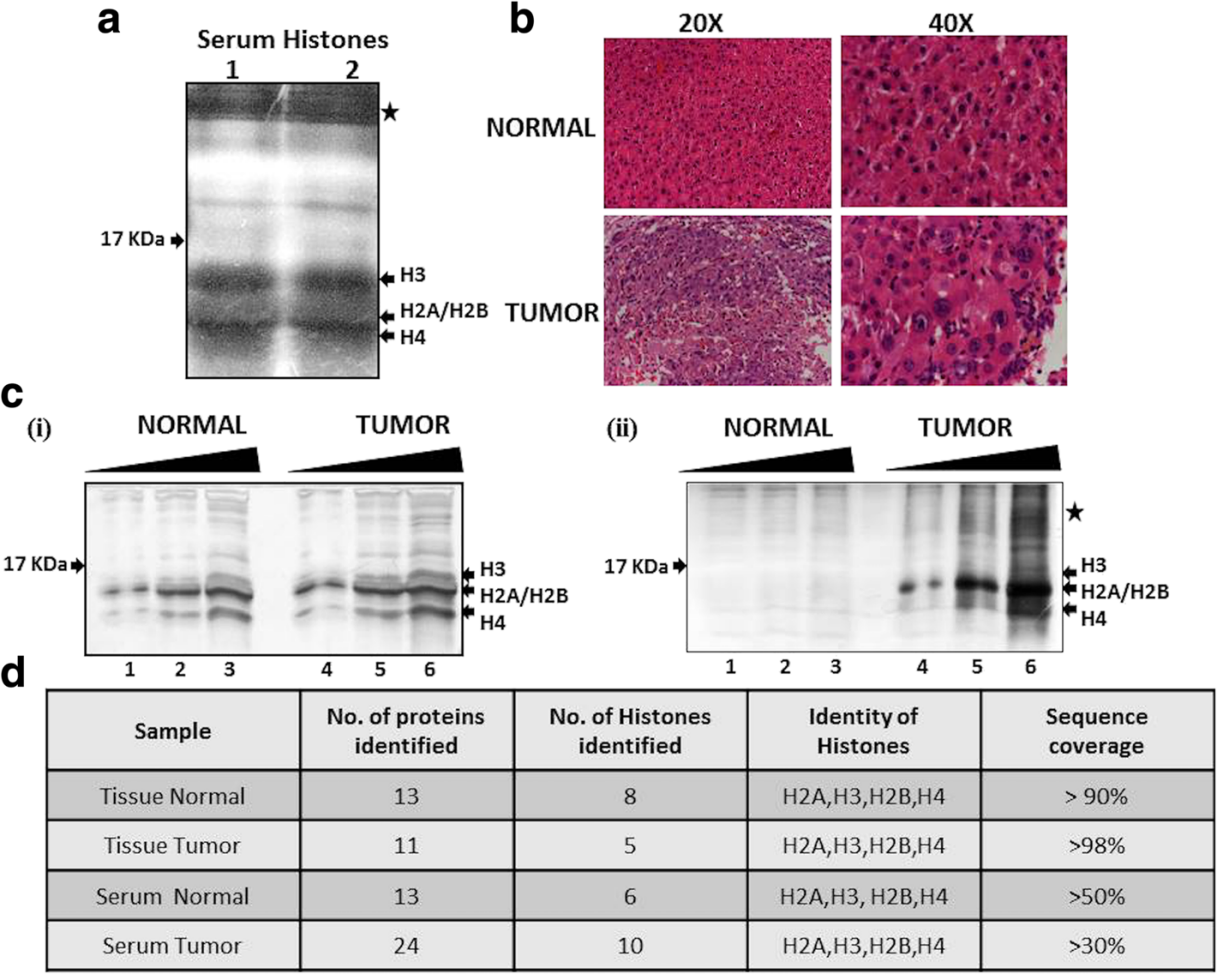
Resolution and identification of purified histones from paired serum and liver tissue of normal and tumour. **a** Silver stained 18% SDS-PAGE confirmed the integrity of the histones isolated from serum samples (1 and 2) of HCC-harboring rats by DAE protocol. All the core histones, H3, H2A, H2B and H4 are marked with an *arrow*, whereas the *star* marks high molecular weight proteins. **b** H&E-stained section of normal liver and HCC showing the altered histology in HCC at 20× and 40× magnification. **c** Silver-stained 18% SDS-PAGE showing the integrity of the purified histones loaded in increasing volumes (5, 10 and 15 μl) (*i*) samples of normal and tumour tissues and (*ii*) serum of normal and tumour bearing rats by the DAE protocol. The core histones H2A, H2B, H3 and H4 are marked. **d** Histones isolated from tissues and serum of both normal and tumour harboring rats are subjected to LC-MS after trypsin digestion. The obtained data on number of peptides of histones identified along with sequence coverage are tabulated and confirmed the identity of purified proteins by DAE method. *H&E* haematoxylin and eosin, *NDEA N*-nitrosodiethylamine

We developed a minimally invasive and cost effective, robust protocol for isolation of histones from serum samples. This method comprises of precipitation of total serum proteins by acid followed by purification of basic proteins by the acid extraction method. As the method involves precipitation and extraction by two acids, it is referred as Dual Acid Extraction (DAE) method. There are four key steps in the protocol: First step is the isolation of serum from the blood; second step is the total protein precipitation by use of trichloroacetic acid (TCA). TCA, unlike other chemicals, precipitates all the proteins irrespective of their molecular weight and is also independent of the physico-chemical properties of proteins; in the third step, histone extraction was carried out by use of the 0.2 M H_2_SO_4_ to separate histones from other proteins; and in the final step, acetone and acidified (hydrochloric acid) acetone were used for removing the traces of TCA or H_2_SO_4_ by replacement of sulphate group (SO_4_^2−^) with chloride group (Cl^1−^) from isolated histones (Fig. 1). The quality of isolated histones was checked by loading on to a 18% SDS-PAGE followed by silver staining. The four core histones—H2A, H2B, H3 and H4—were visualized on the gel, but along with them, other high molecular weight proteins were also noted (Fig. 2a).

To understand whether histone PTM changes observed in the tissue samples can be seen in the histones purified from the serum, we used a rat animal model system. Hepatocellular carcinoma (HCC) was induced in Sprague-Dawley rats by administering *N*-nitrosodiethylamine (NDEA) in drinking water at a concentration of 1 ppm/g body weight. After 120 days of NDEA administration, HCC was confirmed by haematoxylin and eosin (H&E) staining (Fig. 2b). The H &E-stained slides showed classical features of HCC like well-vascularized tumours with wide trabeculae, prominent acinar pattern, cytologic atypia and vascular invasion. Histones were isolated from both the liver tissues and serum of normal and tumour-bearing rats. The histones obtained from serum were quantified by Bradford method using BSA as a standard. The histone quantity in the serum of the HCC-bearing rat group was found to be significantly higher than the normal group (Fig. 2c (ii)). The concentration was 0.8 and 5.4 μg/mL serum for normal and tumour respectively. The purified histones from tissues (Fig. 2c (i)) as well as serum (Fig. 2c (ii)) were resolved on an 18% SDS-PAGE and silver stained to check their integrity. To confirm their identity, the histones were subjected to liquid chromatography-coupled mass spectrometry analysis (LC-MS). All the core histones were identified in both the serum samples (normal and tumour) with query coverage of >50% in case of normal and >30% for tumour (Fig. 2d). Also, of the total 13 proteins identified in serum of normal rats, three correspond to histone H2A and one each to histone H3.1, H2B and H4. In case of the serum of tumour-bearing rats, out of the 24 proteins identified, six were of histone H2A subtypes, two of histone H2B subtypes and one each of H3.1 and H4 histones (Fig. 2d and Table 1). Apart from histones, many other basic serum proteins were also identified in MS, the details of which are presented as a part of the Table 1. Histones isolated from the tissue were used as a positive control in LC-MS, where all the core histones were detected with >90% query coverage in both normal and HCC.

**Table 1:** Total proteins detected in LC-MS of serum and tissue purified histories of normal and tumour samples

Proteins	Tissue normal	Tissue tumour	Serum normal	Serum tumour
Histones	H2A type 1 (91.5%) H2A type 2-A (91.5%) H2A type 1-C (91.5%) H2A type 1-F (86.1%) H2B type 1E (91.5%) H2B type 1C (91.5%) H3.1 (99.2%) H4 (59.2%)	H2A type 2-A (98.4%) H2A type 1-C (98.4%) H2B type 1E (98%) H3.1 (99.2%) H4 (98.4%)	H2A type 2-A (81.5%) H2A type 1 (74.62%) H2A type 1-C (50%) H3.1 (59.2%) H2B type 1E (57%) H4 (60%)	H2A type 2-A (46.9%) H2A type 1-C (33%) H2A type 1-F (38.4%) H2A type 1-E (30%) H2A type 1 (33%) H2A type 4 (33%) H2B type 1E (57%) H2B type 1C (35%) H3.1 (40.7%) H4 (47%)
Non-histones	Prelamin A/C (26%) KDM5D (274%) ESF1 (30.6%) ESF1 homolog (23.9%) Acid-sensing ion channel 5 (12.9%)	Prelamin A/C (62.4%) KDM5D (30.32%) EIF2S3Y (29.8%) ESF1 homolog (41%) Acid-sensing ion channel (17.9%) Ribonuclease UK114	Fibrinogen beta chain (65.75%) Fibrinogen alpha chain (56.91%) Isoform 2 of Fibrinogen alpha (69.45%) Isoform 2 of Fibrinogen beta chain (70.96%) Translation initiation factor elf-2B (46.1%) Fertuin-B (47.6%) Putative pheromone receptor (18.7%)	Fibrinogen beta chain (65.3%) Fibrinogen alpha chain (43.3%) Isoform 2 of Fibrinogen beta chain (67.4%) Isoform 2 of Fibrinogen alpha chain (59.6%) Matrix estracellular phosphoglycoprotein (60%) Potassium voltage-gated channel subfamily C member 2 (30.8%) ESF1 homolog (57.9%) Alkaline phosphatase (57%) Indoleamine 2.3-dioxygenase 1 (37.8%) Aldehyde oxidase 2 (29.6%) Astrocytic phosphoprotein (69.9%) Transcription factor YY2 (32.9%) Cannabinoid receptor 1 (11.1%) Putative pheromone receptor (19.1%)

### Histone PTM pattern is comparable in liver tissues and respective serum samples

**Fig. 3:**
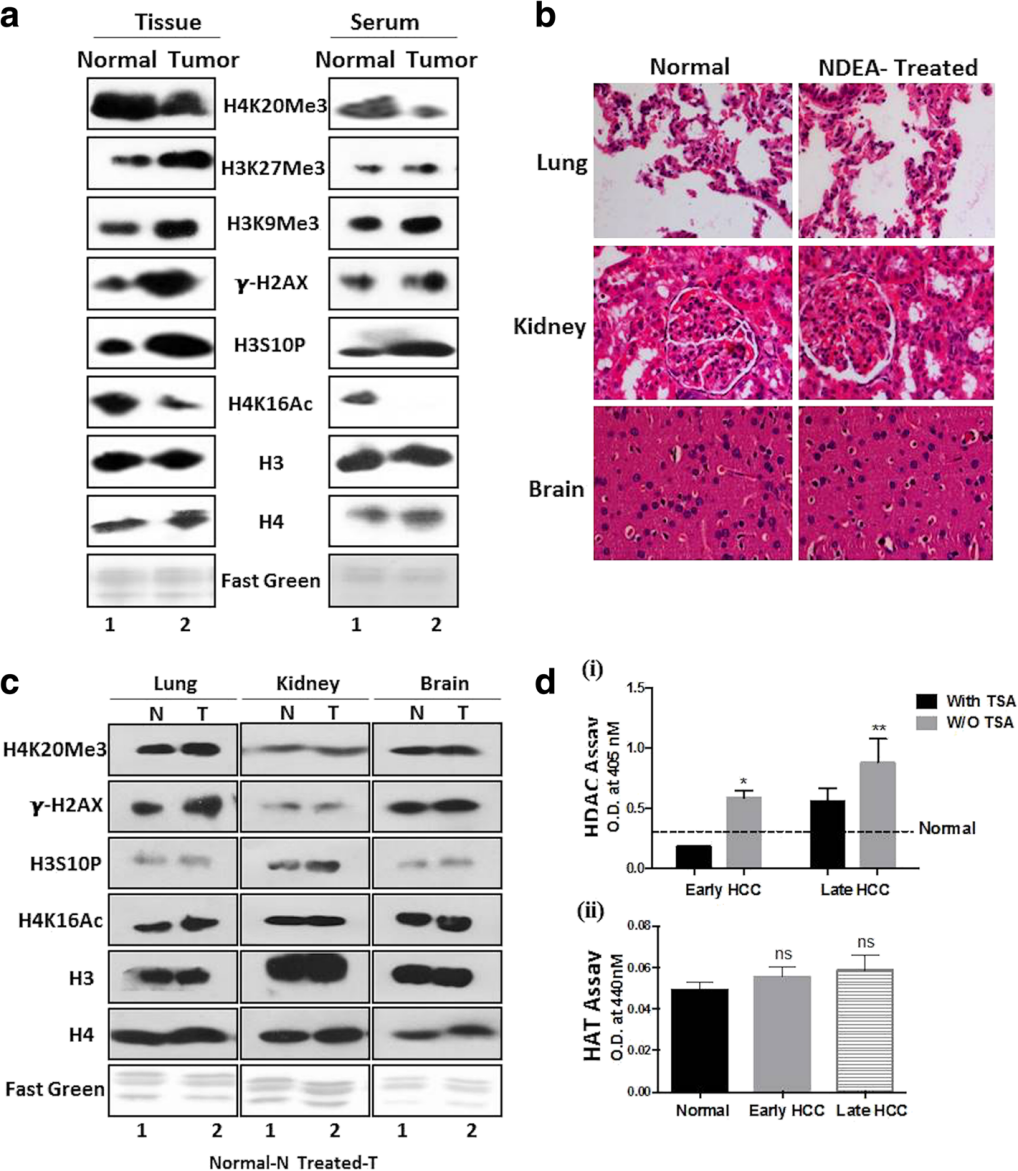
Profiling of site-specific histone modifications and modifiers in paired serum and liver tissue of normal and tumour. **a** (*i*) Western blots of histones isolated from tissues and serum with site-specific histone PTM antibodies of H4K20Me3, H3K27Me3, H3K9Me3, $\boldsymbol{\upgamma}$-H2AX, H3S10P and H4K16Ac in tumour tissue and serum compared to normal. Antibodies against H3 and H4, and fast green-stained histone transferred PVDF membrane were used as equal loading control. **b** H&E-stained section (×40) of tissues (lung, kidney and brain) from NDEA-treated or untreated (normal) animals. **c** Western blotting of histones isolated from normal tissues (lung, kidney and brain) and serum of NDEA-treated or untreated animals with site-specific histone PTM antibodies against H4K20Me3, $\boldsymbol{\upgamma}$-H2AX, H3S10P and H4K16Ac. Antibodies against H3 and H4, and fast green-stained histone transferred PVDF membrane were used as equal loading control. **d** Colorimetry-based assay done using serum isolated from normal, early and late stage tumour bearing rats (*i*) HDAC activity assay was performed on both the TSA-treated and untreated samples (*ii*) HAT activity. Statistical tests are done by using Student’s *t* test. *p < 0.05, **p < 0.01

The histone PTM profiling of normal and HCC tissues was compared with respective serum samples by immunoblotting with site-specific antibodies against H4K16Ac and H4K20Me3, as the loss of these marks are established as hallmark of human cancer [3]. As reported for human tumours, rat tumour tissue has also showed hypo-acetylation and hypo-methylation at H4K16 and H4K20 respectively (Fig. 3a). Interestingly, respective serum histones also showed the same pattern as of tissue histones (Fig. 3a). The other crucial histone PTM marks like γH2AX—a DNA damage mark, H3K27Me3 and H3K9Me3—transcriptional repressive marks and H3S10P—a mitotic mark, known to be deregulated in many of the human cancers [21, 22] were also probed. Intriguingly, we noted a significant increase in these marks in both the tumour sample tissue and serum (Fig. 3a). This shows that the key histone modifications like acetylation, methylation and phosphorylation are retained during the course of histone extraction from serum samples by DAE method and also profiling between tissue samples and their respective serum samples mirrored each other.

In order to understand that the changes seen in serum histone is due to liver tumour and not due to changes in the entire PTM profile of all the other organs, we profiled for histone PTMs in the lung, kidney and brain isolated from both the normal and NDEA-treated (tumour-bearing) animals. The tissues isolated from the animals treated with NDEA were first histologically examined for any aberrations by H&E staining (Fig. 3b). Grossly, no differences were seen with respect to control tissues in untreated animals. Histones were then isolated and probed for site-specific PTM antibodies (Fig. 3c). The data demonstrates that the alterations of modifications are specific to tumour liver tissue and not to other histologically normal organs, thus strengthening that the liquid biopsy (serum histone) is very similar to its parent tissue (tumour tissue-liver) and hence can be used for clinical purpose.

### Increased HDAC activity in HCC tissues correlates with corresponding serum samples

So far, we have seen a positive correlation for histone PTMs in HCC, tissue and serum histones. We next contemplated whether the reported increased HDAC activity in tumour tissue [23] can also be seen in serum to understand how similar the liquid biopsy (serum) is to tissue biopsy in context of histone PTM modifiers. This is particularly interesting due to the observation of H4 hypo-acetylation seen in serum and HCC purified histones. HDAC activity assay conducted using serum isolated from normal, early (90 days NDEA treatment) and late stage (120 days NDEA treatment) HCC-bearing rats demonstrated the presence of significantly higher HDAC activity in both the tumour serum samples and normal serum (Fig. 3d (i)). Interestingly, the serum of early stage liver cancer animals also showed an elevated HDAC in comparison to normal, indicating that HDAC activity gradually increases during the course of HCC development (Fig. 3d (i)). As a proof of principle that the calorimetric assay performed is indeed a true measure of HDAC activity, treatment of serum samples with TSA was done prior to HDAC assay. As seen in (Fig. 3d (i)), the activity in samples treated with TSA is less compared to the untreated samples validating the robustness of the assay. To understand the status of HATs, levels of which are also reported to change in human tumours [24], we performed a HAT activity assay in normal, early and late stages of tumour serum, but no significant differences were observed, suggesting the H4 hypo-acetylation seen might be due to enhanced HDAC activity in tumour samples compared to normal (Fig. 3d (ii)).

**Fig. 4:**
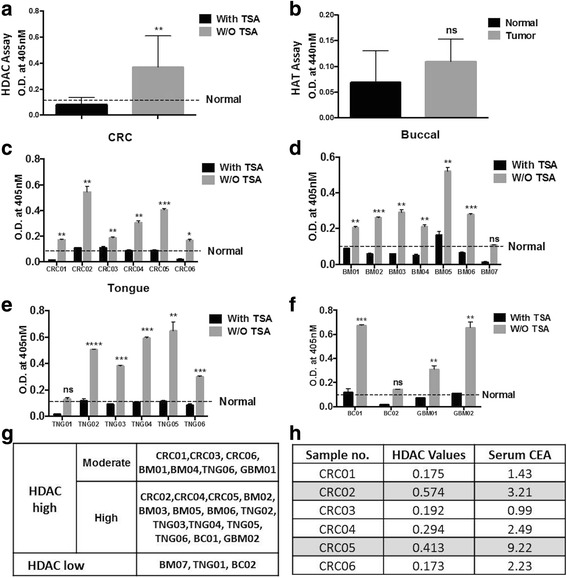
Profiling of histone modifiers in human serum samples. Cumulative analysis of **a** HDAC and **b** HAT activity assays done using the human serum of six healthy subjects and 24 cancer patients. With TSA—involves serum pre-treated with TSA for 10 min and without TSA—untreated samples. Graph showing the relative activity of HDACs in **c** CRC, **d** buccal, **e** tongue, **f** breast and glioblastoma samples. Statistical tests are done by using Student’s *t* test. *p < 0.05, **p < 0.01. **g** Table representing the subgrouping of all 24 human samples as per their HDAC activities. **h** Correlation between serum CEA levels and HDAC activity in six CRC samples. *Highlighted samples* show high CEA and HDAC levels

Intrigued by the observation of elevated HDAC activity in tumour serum samples of rat model, we asked whether the HDAC activity can also be detected in human serum samples. To this end, we performed HDAC activity assay in serum isolated from six healthy subjects (normal) and 24 different types of cancer patients, including colorectal (CRC#6), buccal (BM#7), tongue (TNG#6), breast (BC#2) and glioblastoma (GBM#2). The histopathological details of patient samples included in the study are tabulated in Table 2. The cumulative analysis of all the 24 samples revealed a higher HDAC activity in serum of cancer patients compared to normal (Fig. 4a) with no significant change in HAT activity (Fig. 4b). Also, grouping of serum samples based on the cancer type shows an elevated activity in most of the samples of CRC (Fig. 4c), buccal (Fig. 4d), tongue (Fig. 4e), and breast and glioblsatoma (Fig. 4f).

**Table 2:** Histopathological analysis of human patient samples used in the study

Sample	Origin	Histopathological analysis
BM01	Buccal	Moderately differentiated squamous carcinoma
BM02	Buccal	Moderately differentiated squamous carcinoma
BM03	Buccal	Moderately differentiated squamous carcinoma
BM04	Buccal	Moderately differentiated squamous carcinoma
BM05	Buccal	Moderately differentiated keratinizing squamous carcinoma
BM06	Buccal	Moderately differentiated keratinizing squamous carcinoma
BM07	Buccal	Poorly differentiated squamous carcinoma
TNG01	Tongue	Moderately differentiated squamous carcinoma
TNG02	Tongue	Moderately differentiated squamous carcinoma
TNG03	Tongue	Moderately differentiated squamous carcinoma
TNG04	Tongue	Squamous carcinoma
TNG05	Tongue	Moderately differentiated squamous carcinoma
TNG06	Tongue	Moderately differentiated squamous carcinoma
CRC01	Colon	Moderately adenocarcinoma
CRC02	Colon	Poorly differentiated adenocarcinoma
CRC03	Colon	Moderately differentiated adenocarcinoma
CRC04	Colon	Moderately differentiated adenocarcinoma
CRC05	Rectum	Moderately differentiated adenocarcinoma
CRC06	Rectum	Adenocarcinoma
BC01	Breast	Infitrating duct carcinoma, Grade II
BC02	Breast	Infitrating lobular carcinoma, Grade II
GBM01	Glioblastoma	Glioblastoma (WHO grade IV)
GBM02	Glioblastoma	Glioblastoma (WHO grade IV)

Interestingly, we found that even with a sample size of 24, the HDAC activity is different amongst each patient sample, therefore, highlighting the importance of subgrouping the patients on the basis of inherent epigenetic background for success of epigenetic drugs (epi-drug) therapy. Thus, monitoring the HDAC activity in the serum sample has helped us to categorize the patients into two subgroups: (1) high HDAC and (2) low HDAC (Fig. 4g). Group 1 can further be subdivided into two, high and moderate HDAC activity groups (Fig. 4g). This subgrouping of patients on the basis of HDAC activity will assist in selection of patients, determining the dose of epi-drug, thus increasing the success of therapy.

During the diagnosis of a disease like cancer, measurement of many serum based biomarkers is usually performed. One such marker is the quantification of serum CEA levels in CRC patients. We observed differential levels of CEA amongst the six CRC samples used in the study. Interestingly, high CEA levels seen for CRC02 and CRC05 samples correlated with high HDAC activity in comparison to other samples with low CEA values (Fig. 4h). Thus, serum based measurement of HDAC activity compares positively to CEA, a cancer biomarker for CRC.

## Discussion

The DAE protocol developed for the isolation of histones from serum samples is simple, robust, time efficient and cost effective. Further, the comparison of key site-specific histone PTMs in tissue versus serum, for both normal and tumour samples, has revealed a similar pattern amongst the respective biological samples. Also, we illustrate for the first time a higher HDAC activity in serum tumour sample compared to normal.

Earlier protocols for isolation of cNUCs are based on chromatin immunoprecipitation (ChIP) by specific histone antibodies. ChIP depends critically on the quality of the antibody and is not cost-effective. Though numerous histone antibodies as ChIP-grade are available, they need stringent validation by western blotting and MS, because of their cross-reactivity with unmodified histones, similar histone modifications and some with non-histone proteins. The developed protocol overcomes all the limitations of the existing ChIP method as it is based on dual acid extraction. The good quality and quantity of purified histones by this novel technique has opened a new avenue for studying the pattern of histone PTMs as well as histone variants in serum samples by western blotting or mass spectrometry in different pathophysiological states.

To gain insights into global serum histone PTM pattern and understand whether the observed alteration matches with that of the cancer tissue, NDEA-induced HCC rat model system has been used. The quantity of histones isolated from the normal serum was very less compared to the serum samples of NDEA-induced HCC rats (Fig. 2c (ii)). This could be directly attributed to the increased cell death seen in diseased conditions as opposed to the healthy subjects, due to which the abundance of cNUCs and hence histones will be more in a serum sample of diseased state. This is in conformity with the earlier reports where elevated cNUCs are observed in various human cancers [6, 10, 12, 25].

Studies on global histone modifications across various human cancers have identified alterations in a panel of histone PTMs [26]. The loss of H4K16Ac and H4K20Me3 are now regarded as the hallmark of most human cancers [3]. Interestingly, we noted that these hallmarks are even true for the animal model system. Furthermore, similar changes are observed in serum histones also proving that indeed they are true hallmarks which change during cancer, irrespective of the cancer type in the study, species used and source of biological samples (liquid or solid biopsy). Profiling for other histone PTMs—H3K27Me3, H3K9Me3, γH2AX and H3S10P, in both tissue and serum showed a same pattern of increase in tumour tissue and serum samples. Further, we also reveal that the changes in the histone PTM pattern observed in serum mirrors only liver tissue and no other organs, thus proving that the histones in serum are from tumour tissue.

Earlier studies had shown the presence of acetylated histones in blood cells, but its levels were not correlated with tumour tissue [27]. Such studies on global histone modifications are of importance because of their prognostic utility and predictive markers for recurrence. Now with the serum histones showing a similar pattern of histone PTMs as of tumour tissue, liquid biopsy (non-invasive) will be a good alternative to tissue biopsy (invasive) for monitoring the disease regression/progression in cancer patients. Further, the antitumour effects of histone deacetylase or histone methyltransferase inhibitors could be evaluated by monitoring changes in the quantities of the corresponding modification on the circulating histones thus overcoming the limitation of earlier studies wherein histones of blood cells were used.

Altered histone acetylation levels in cancer are the result of the imbalance of the activities of HAT and HDAC. Many reports have emphasized the altered levels of these enzymes, especially HDACs in various cancers [28]. Our data of an increased HDAC activity in serum and tumour tissue samples compared to normal is in accordance with the earlier reports. Thus, the present observation of HDAC activity in serum allows us to monitor the level of HDAC and possible tumour status in response to histone-modifying enzyme inhibitors in real time by liquid biopsy. Interestingly, some studies on global proteomic profiling of serum proteins has earlier reported the presence of HDACs in serum, thus it is not surprising that HDAC activity is detected in serum [29, 30]. Intriguingly, measurement of HDAC activity in early (90 days NDEA treatment) and late (120 days NDEA treatment) stages of liver cancer in an animal model system revealed a gradual increase in HDAC activity as the animal develops a tumour. This observation proves that early monitoring of HDAC activity may assist in better diagnosis of cancer. Further, the assay can be employed to monitor the naive cancer, recurring and drug resistant tumours, where in periodic monitoring of HDAC activity in patients diagnosed with cancer will aid in their improved clinical management. However, large studies including more cancer patients at various stages, undergoing diverse chemotherapeutic treatments in combination with epi-drugs will provide conclusive evidences and help in employing the proposed hypothesis for better disease management.

**Fig. 5:**
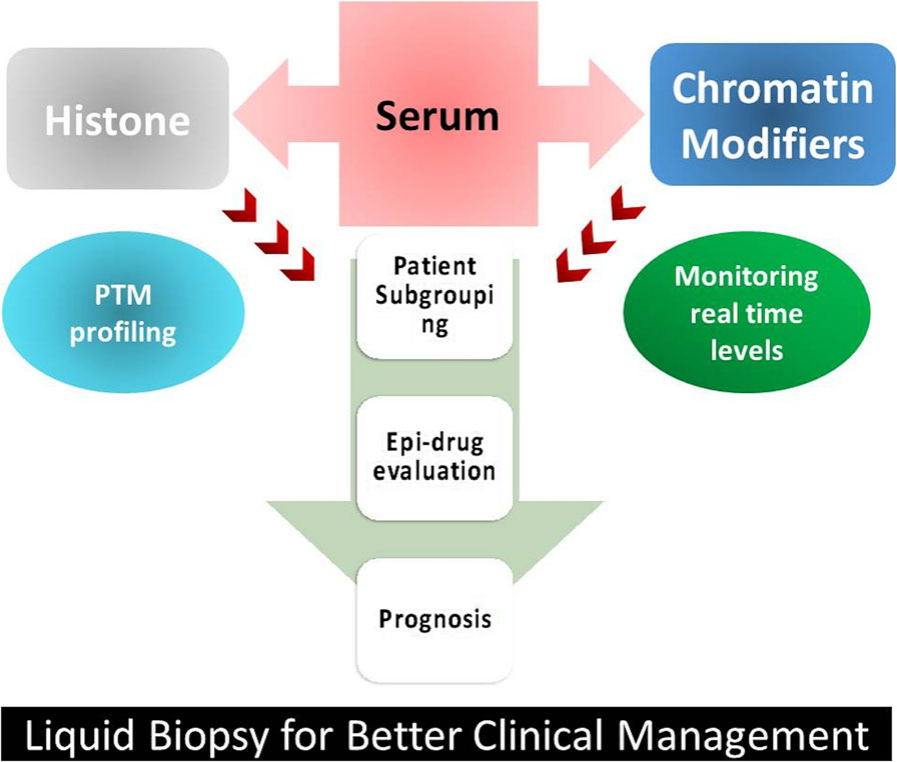
Schematic representation of liquid biopsy as tool for better clinical management of cancer patients. The DAE protocol provides ‘real-time’ purification of serum histones and monitoring of levels of chromatin modifiers like HDACs and HATs that will help in subgrouping patients eligible for epi-drug treatment and their response, aid in better prognosis and disease management

Owing to the increased HDACs, use of HDAC inhibitors for treatment of cancer is considered to be a good treatment regime. But so far, the use of HDAC inhibitors had limited success in solid tumours because of severe toxicities and other fatal effects [31]. One of the reasons behind the failure or limited success of HDAC inhibitors has been attributed to the inherent epigenetic background of the patients, where some may have high expression of HDACs others low levels [32, 33]. This can further be seen in our data, where all the patients showed different extent of HDAC levels (Fig. 4c, f). Therefore, administering HDAC inhibitors to patients with inherent low levels of HDACs may be fatal. For example, based on our data, we have segregated the samples into two groups, HDAC high and HDAC low (Fig. 4g). Treating the first group patients with HDAC inhibitors will be beneficial, as this group show elevated levels of these enzymes. Furthermore, this group can be subdivided into two, which can help in deciding the dose and time span of epi-drug therapy, where a high HDAC activity patient can be given high doses of drugs for a longer span than the others. As for the second group, epi-drug treatment might not be of much benefit. Thus, serum-based detection of histone modifiers like HDAC/HAT will help with subgrouping of patients and personalize the treatment, monitor patient’s health status during treatment with histone modifier inhibitors and response of the individual’s tumour to treatment (Fig. 5).

## Conclusions

In summary, the developed DAE technique for isolating serum histones will provide real time information of epigenome which will be helpful in understanding the overlap between paired serum and cancer tissue histone proteome by high-end proteomics. Importantly, our data gave a novel rationale for using serum histone proteomics as a predictive tool for disease progression. This information will allow the development of efficient strategies for the treatment and better management of the underlying disease. The combination of different histone marks rather than a single histone mark is believed to further enhance sensitivity and specificity of detection of these marks and hence improve cancer management.

## Abbreviations

cNUCs: Circulating nucleosomes
Epi-drugs: Epigenetic drugs
HAT: Histone acetylase
HCC: Hepatocellular carcinoma
HDAC: Histone deacetylase
